# Childhood trauma and psychological symptoms in young adults: examining the mediating role of vulnerable narcissism and the moderating role of psychological resilience

**DOI:** 10.3389/fnbeh.2026.1904564

**Published:** 2026-09-16

**Authors:** Fatih Cebeci

**Affiliations:** 1 Department of Social Work, Faculty of Health Sciences, Istanbul Medipol University, Istanbul, Türkiye; 2 UNEC Social Work and Social Innovations Research Center, Azerbaijan State University of Economics, Baku, Azerbaijan

**Keywords:** anxiety, childhood trauma, depression, psychological resilience, stress, vulnerable narcissism

## Abstract

**Introduction:**

Childhood trauma is consistently associated with symptoms of depression, anxiety, and stress, yet the self-related processes and adaptive resources relevant to these associations remain insufficiently understood. This study examined whether vulnerable narcissism statistically mediated the associations between childhood trauma and these three symptom domains and whether psychological resilience moderated the associations between vulnerable narcissism and these symptoms.

**Methods:**

A cross-sectional convenience sample of 571 young adults aged 18–30 years was recruited online from Türkiye. Of the participants, 76.7% were women, and the sample largely comprised individuals engaged in university education. Participants completed the Childhood Trauma Questionnaire-33, the eight-item Vulnerable Narcissism Scale, the Brief Resilience Scale, and the Depression Anxiety Stress Scales-21. Regression-based mediation and moderated mediation analyses were conducted using 5,000 bootstrap samples and heteroscedasticity-consistent standard errors.

**Results:**

Childhood trauma and vulnerable narcissism were positively associated with depression, anxiety, and stress symptoms, whereas psychological resilience was negatively associated with all three symptom domains. The indirect associations between childhood trauma and depression, anxiety, and stress through vulnerable narcissism were statistically significant, while the corresponding direct associations remained significant. However, psychological resilience did not moderate the associations between vulnerable narcissism and any of the three symptom domains or the indirect associations through vulnerable narcissism.

**Discussion:**

The findings support partial statistical mediation through vulnerable narcissism within the specified model but do not establish a temporal, developmental, or causal sequence. Psychological resilience was independently associated with lower symptom levels but did not function as a moderator in the proposed model.

## Introduction

Childhood trauma encompasses multiple forms of abuse and neglect (Bernstein et al., 1994) and is associated with psychological difficulties that may persist into adulthood. Meta-analytic evidence indicates elevated odds of adult depression among individuals exposed to childhood maltreatment (Mandelli et al., 2015), while a broader synthesis of adverse childhood experiences found higher odds of both depression and anxiety among adults reporting four or more adversities than among those reporting none (Hughes et al., 2017). A similar pattern has been documented in Türkiye, where adverse childhood experiences were positively associated with symptoms of depression, anxiety, and stress among university students aged 18–25 years (Girgin et al., 2025). These associations may extend beyond psychological symptoms to difficulties in self-related functioning. From attachment and schema perspectives, adverse or maladaptive caregiving experiences are associated with insecure internal working models of relationships, a fragile sense of self, and self-esteem that depends heavily on external approval (Otway and Vignoles, 2006; Young et al., 2003). These characteristics overlap with vulnerable narcissism, a broader personality pattern characterized by hypersensitivity, anxiety, interpersonal sensitivity, shame, and contingent or fluctuating self-esteem (Hendin and Cheek, 1997; Miller et al., 2011; Pincus et al., 2009). This conceptual overlap makes vulnerable narcissism particularly relevant to understanding the self-related dimension of the association between childhood trauma and psychological symptoms. Building on this framework, the present study examined vulnerable narcissism as a self-related personality correlate and psychological resilience as an adaptive resource in the associations of childhood trauma with symptoms of depression, anxiety, and stress among young adults.

### Childhood trauma, psychological symptoms, and the mediating role of vulnerable narcissism

Positive associations of childhood trauma and related forms of childhood adversity with symptoms of depression, anxiety, and stress have been documented across different samples. Among Turkish university students, childhood psychological maltreatment was associated with depressive symptoms through indirect pathways involving insecure attachment, guilt, and loneliness (Guler, 2022). Similarly, adverse childhood experiences were positively associated with depression, anxiety, and stress among Turkish emerging adults, with psychological resilience identified as a statistical mediator of these associations (Girgin et al., 2025). Related findings were reported among nurses, for whom emotional abuse and emotional neglect were independently associated with all three symptom domains (Gürsoy and Mechmet, 2023). In another university sample, depression, anxiety, and stress statistically mediated the association between childhood trauma and psychological vulnerability (Yıldız and Yukay Yüksel, 2024). On the basis of this evidence, positive associations were expected across all three symptom domains.

Hypothesis 1: Childhood trauma is significantly and positively associated with symptoms of depression, anxiety, and stress.

Research on the association between childhood trauma and psychological symptoms has considered several emotional, interpersonal, and cognitive processes. These have included emotion-regulation difficulties (Alpay et al., 2017), perceived social support and emotion-regulation difficulties (Huang et al., 2026), negative beliefs about emotion and psychological inflexibility (Taşören, 2023), and maladaptive cognitive emotion-regulation strategies (Huh et al., 2017). Self-compassion has also been examined in related attachment–psychopathology models (Set, 2021). Whereas these constructs capture relatively specific emotional, cognitive, or relational processes, vulnerable narcissism represents a broader personality pattern involving fragile and contingent self-esteem, hypersensitivity to evaluation, shame-proneness, interpersonal withdrawal, and dependence on external validation. It is also empirically distinguishable from grandiose narcissism (Hendin and Cheek, 1997; Miller et al., 2011; Pincus et al., 2009). Vulnerable narcissism was therefore selected as the focal mediator in the present study.

This conceptual rationale has been complemented by empirical evidence linking vulnerable narcissism to trauma-related difficulties in self-functioning. Network analysis identified entitlement rage and contingent self-esteem as central features of vulnerable narcissism; emotional maltreatment showed stronger connections with vulnerable narcissistic features than did other forms of maltreatment, and attachment security emerged as a bridge between the two domains (Wang et al., 2023). Vulnerable narcissism and self-impairment also formed a central dyad across trauma profiles (Benzi et al., 2026). Meta-analytic evidence similarly indicated that childhood maltreatment was more strongly associated with vulnerable than with grandiose narcissism, although the associations with both forms were small. Neglect also showed a stronger association with vulnerable narcissism than did physical abuse (Gao et al., 2024).

Attachment and schema perspectives have provided a framework for interpreting this pattern. Parental coldness and excessive admiration were associated with overt and covert narcissism, whereas attachment anxiety was specifically associated with covert narcissism (Otway and Vignoles, 2006). Schema theory further proposes that adverse caregiving experiences may contribute to schemas involving defectiveness, dependence, and approval-seeking (Young et al., 2003). Evidence from Turkish young adults was also consistent with these perspectives: vulnerable narcissism was associated with preoccupied attachment and depression, while depression was associated with emotional neglect and overprotection–overcontrol (Şar and Türk-Kurtça, 2021). Across these theoretical and empirical perspectives, adverse caregiving experiences may be associated with difficulties in maintaining stable, internally grounded self-worth.

Evidence more directly relevant to the proposed indirect pathway has come from cross-sectional indirect-effect models. Significant indirect effects were reported through vulnerable narcissism and emotion dysregulation in the association between childhood emotional abuse and binge-watching (Verrastro et al., 2024). Kealy et al. (2023) reported indirect effects of childhood emotional neglect on depressive and generalized anxiety symptoms through narcissistic vulnerability alone and through the sequential pathway from narcissistic vulnerability to shame. Related evidence indicated that individuals with social anxiety disorder reported higher levels of childhood trauma and vulnerable narcissism than healthy controls and that both constructs were positively associated with social anxiety symptom severity; however, mediation was not examined in that study (Gökçay et al., 2024). In a non-clinical adult sample, significant partial indirect effects through vulnerable and grandiose narcissism were also observed in the association between childhood abuse or neglect and prodromal psychosis symptoms (Blundell and van Doorn, 2025). Despite this evidence, the statistical mediating role of vulnerable narcissism in the associations of childhood trauma with depression, anxiety, and stress has not been examined across all three symptom domains within the same non-clinical young adult sample. This gap informed Hypothesis 2.

Hypothesis 2: Vulnerable narcissism statistically mediates the association between childhood trauma and symptoms of depression, anxiety, and stress.

### The moderating role of psychological resilience

Resilience has been conceptualized both as a trajectory of adaptation and as an individual capacity. Bonanno (2004) described posttraumatic resilience as a pattern of relatively stable healthy functioning and distinguished it from recovery following an initial period of disruption. For the present study, however, a narrower operational definition has been adopted. The Brief Resilience Scale (BRS) assesses the perceived ability to recover or bounce back from stress (Smith et al., 2008). Resilience has therefore been treated as a self-reported recovery capacity rather than as a longitudinal trajectory of adaptation. Consistent with this distinction, the hypotheses have been grounded in the protective model, in which a resource attenuates the association between risk and an adverse outcome through an interaction. This model differs from the compensatory model, in which a promotive factor is independently associated with the outcome (Fergus and Zimmerman, 2005). Evidence for this buffering role has been mixed. In a two-wave study of Chinese adolescents, resilience moderated only the association between a high-neglect profile and subsequent internalizing problems; comparable interactions were not observed for other maltreatment profiles or externalizing problems (Cao et al., 2024). Among adults, resilience was negatively associated with depressive symptom severity and moderated the association between non-childhood trauma and depression, whereas the childhood abuse × resilience interaction was not statistically significant (Wingo et al., 2010). Beyond moderation, resilience has also been examined within alternative statistical pathways. In a cross-sectional clinical sample, resilience and daily stress partially mediated the association between childhood trauma and depression, including a sequential indirect path from resilience to daily stress (Zheng et al., 2022). These differing results indicate that the statistical role of resilience may vary according to the adversity, psychological outcome, population, and pathway examined.

The placement of resilience at the vulnerable narcissism–symptom stage has been supported indirectly by findings concerning the stress-related functioning of vulnerable narcissism. Pathological narcissism has been associated with shame and interpersonal distress, while its vulnerable dimensions have been linked particularly to socially avoidant interpersonal problems (Pincus et al., 2009). In addition, vulnerable narcissism has been associated with denial and greater behavioral disengagement under stress after controlling for trait anxiety and social desirability (Fernie et al., 2016). Consistent with this pattern, vulnerable narcissism has been negatively associated with all three dimensions of resilience, with the strongest association observed for engineering resilience, which concerns recovery and stabilization following stressful disruption (Sękowski et al., 2023). In the third study reported by Papageorgiou et al. (2019), vulnerable narcissism was negatively associated with mental toughness and positively associated with perceived stress. Meta-analytic evidence has further indicated a positive association between vulnerable narcissism and self-reported negative mental health outcomes (Hou et al., 2025). Although these findings link vulnerable narcissism with difficulties in recovering from stress, none of the cited studies directly tested whether resilience moderated its association with psychological symptoms. In the absence of direct tests, resilience has been positioned as a second-stage moderator to assess whether the associations between vulnerable narcissism and current symptoms of depression, anxiety, and stress vary according to the recovery capacity measured by the BRS. Because the BRS was designed to assess the perceived ability to recover from stress (Smith et al., 2008), it does not provide a retrospective measure of resilience during childhood; accordingly, resilience was not positioned within the childhood adversity–vulnerable narcissism association. Given the context-dependent findings reviewed above, the proposed interaction has been treated as an empirical question, with its expected pattern specified in Hypotheses 3 and 4.

Hypothesis 3: Psychological resilience moderates the association between vulnerable narcissism and symptoms of depression, anxiety, and stress, such that the association is weaker among individuals with higher resilience.

Hypothesis 4: The indirect association between childhood trauma and symptoms of depression, anxiety, and stress through vulnerable narcissism varies across levels of psychological resilience, such that the indirect association is weaker at higher levels of resilience.

### The present study

Childhood trauma has been associated with depression in adulthood (Mandelli et al., 2015) and with both depression and anxiety (Hughes et al., 2017; Kuzminskaite et al., 2022), as well as with depression, anxiety, and stress among Turkish emerging adults (Girgin et al., 2025). Although several explanatory processes have been examined in trauma-related research and related psychopathology models (Alpay et al., 2017; Huh et al., 2017; Kealy et al., 2023; Set, 2021; Young et al., 2003), vulnerable narcissism, as a broader self-related personality pattern, has received less attention. Relevant studies have largely involved clinical samples or narrower outcomes (Kealy et al., 2023; Gökçay et al., 2024), leaving its role across depression, anxiety, and stress in non-clinical young adults unclear. Findings concerning resilience as a buffer have varied by adversity and outcome (Cao et al., 2024; Wingo et al., 2010; Mutiso et al., 2025). Although Hu et al. (2025) found that resilience moderated the neuroticism–depression association in an analogous second-stage model, its role in the vulnerable narcissism–symptom association remains unclear. Against this background, the present study tested a second-stage moderated mediation model specifying vulnerable narcissism as the statistical mediator of childhood trauma–symptom associations and psychological resilience as the moderator of vulnerable narcissism–symptom associations (Figure 1). The selected age range broadly overlapped with emerging adulthood, a period characterized by changes in identity, autonomy, and interpersonal relationships (Arnett, 2000). Within the Turkish context, overprotection–overcontrol has also been examined alongside depression, vulnerable narcissism, and attachment-related functioning among young adults (Şar and Türk-Kurtça, 2021). Given the cross-sectional design, the specified ordering reflected hypothesized statistical relationships rather than an established temporal or developmental sequence.

**FIGURE 1:**
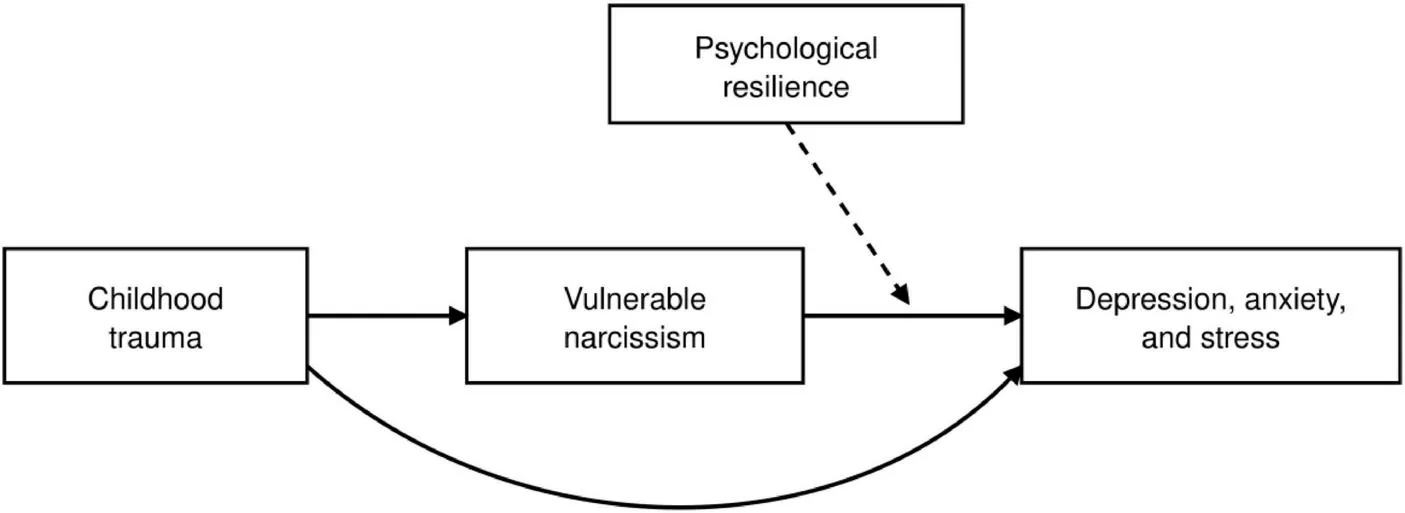
Conceptual model of the relationships among childhood trauma, vulnerable narcissism, psychological resilience, and depression, anxiety, and stress.

## Materials and methods

### Research design

This study employed a quantitative, cross-sectional correlational design to examine the relationships among childhood trauma, vulnerable narcissism, psychological resilience, and symptoms of depression, anxiety, and stress (Büyüköztürk et al., 2009). The mediating role of vulnerable narcissism and the moderating role of psychological resilience were tested within a single statistical model. Given the cross-sectional design, the findings were interpreted as statistical associations rather than evidence of temporal ordering or causality.

### Participants

The study group consisted of 571 young adults aged 18–30 residing in Türkiye. Participation was voluntary, and convenience sampling was used to recruit the sample. The online survey link was distributed through WhatsApp, Instagram, LinkedIn, and e-mail. Recruitment was not based on provincial quotas or regional stratification, and province of residence was not recorded as a study variable. Therefore, the precise geographical coverage and regional representativeness of the sample could not be determined. Convenience sampling was considered appropriate given the practical difficulties associated with reaching young adults aged 18–30 through probability-based sampling procedures (Coşkun et al., 2017). Based on the 2025 Address Based Population Registration System results, the population aged 18–30 years in Türkiye was estimated to be approximately 16.73 million (Turkish Statistical Institute [TurkStat], 2026). Because the data were reported in 5-year age groups, the populations aged 18–19 and 30 years were estimated proportionally. According to Sekaran (2003), a sample of 384 is considered sufficient for a population of this magnitude. The final analyses were conducted with 571 participants, exceeding the recommended minimum sample size. Of the participants, 438 (76.7%) were women and 133 (23.3%) were men. The mean age of the participants was 21.99 years (SD = 3.14). The sample was predominantly composed of university students, and this characteristic was considered when interpreting the findings.

### Data collection tools

#### Sociodemographic information form

A sociodemographic information form was developed by the researcher to obtain information on participants’ gender, age, marital status, educational level, study/employment status, previous psychological support, and psychiatric medication use. The form was developed based on the relevant literature.

#### Childhood Trauma Questionnaire (CTQ-33)

The Childhood Trauma Questionnaire (CTQ-33) was used to assess childhood trauma experiences. The scale was originally developed by Bernstein et al. (1994), and its expanded 33-item version was adapted into Turkish by Şar et al. (2021). The Turkish validation study supported a six-factor structure comprising emotional abuse, physical abuse, sexual abuse, emotional neglect, physical neglect, and overprotection/overcontrol. The scale also demonstrated satisfactory internal consistency and test–retest reliability and differentiated psychiatric and non-clinical groups (Şar et al., 2021). The CTQ-33 consists of 33 items rated on a five-point Likert-type scale (1 = Never, 5 = Very often) and includes six subscales: emotional abuse, physical abuse, sexual abuse, emotional neglect, physical neglect, and overprotection/control. Subscale and total scores are obtained by summing the relevant items. The scale also includes reverse-coded items and a minimization/denial index ranging from 0 to 3, which were considered during data analysis. In the present study, the Cronbach’s alpha coefficient of the scale was 0.92. In the present study, the CTQ-33 total score was used in the hypothesis tests to represent the overall severity of childhood trauma across the six domains. The minimization/denial items were not included in this total score because they function as validity indicators rather than substantive trauma items. The use of the total score was consistent with the construct-level purpose of the proposed model, which focused on the associations of overall childhood trauma severity with vulnerable narcissism and internalizing symptoms rather than on the relative contributions of individual trauma subtypes.

#### Vulnerable Narcissism Scale (VNS)

The Vulnerable Narcissism Scale (VNS), originally developed by Hendin and Cheek (1997), was used to assess vulnerable narcissism. The Turkish adaptation study supported a single-factor, eight-item version of the scale after two items from the original form were excluded because of insufficient factor loadings. All retained items had factor loadings above 0.30, and the internal consistency coefficient was reported as 0.66 (Şengül et al., 2015). Items are rated on a five-point Likert-type scale ranging from 1 (Not at all descriptive/not true/strongly disagree) to 5 (Highly descriptive/true/strongly agree). Higher scores indicate higher levels of vulnerable narcissism, and the total score is obtained by summing item scores. In the present study, the Cronbach’s alpha coefficient of the scale was 0.76.

#### Brief Resilience Scale (BRS)

Psychological resilience was assessed using the Brief Resilience Scale (BRS), originally developed by Smith et al. (2008) and adapted into Turkish by Doğan (2015). The scale consists of six items rated on a five-point Likert-type scale ranging from 1 (Strongly disagree) to 5 (Strongly agree). Items 2, 4, and 6 are reverse coded, and higher total scores indicate greater levels of psychological resilience. Exploratory and confirmatory factor analyses conducted with 295 Turkish university students supported the original single-factor, six-item structure of the scale. The confirmatory factor analysis produced satisfactory fit indices (χ^2^/df = 1.83, CFI = 0.99, RMSEA = 0.05, SRMR = 0.03), and Cronbach’s alpha was 0.83 (Doğan, 2015). In the present study, the internal consistency coefficient of the scale was 0.79.

#### Depression Anxiety Stress Scales-21 (DASS-21)

The Depression Anxiety Stress Scales-21 (DASS-21), originally developed by Lovibond and Lovibond (1995), was used to assess symptoms of depression, anxiety, and stress. The Turkish validation study supported the correlated three-factor structure of the instrument in both community and clinical samples. Confirmatory factor analysis yielded acceptable fit in the community sample (CFI = 0.905, RMSEA = 0.065, SRMR = 0.067) and good fit in the clinical sample (CFI = 0.956, RMSEA = 0.044, SRMR = 0.046), with standardized factor loadings ranging from 0.42 to 0.72 (Sarıçam, 2018). In the adaptation study, Cronbach’s alpha coefficients for the community sample were reported as 0.85, 0.80, and 0.77 for the depression, anxiety, and stress subscales, respectively, whereas corresponding coefficients in the clinical sample were 0.87, 0.85, and 0.81. The scale consists of 21 items rated on a four-point Likert-type scale ranging from 0 (Never/None of the time) to 3 (Always), with seven items in each subscale. Subscale scores are calculated by summing relevant item scores, with higher scores indicating greater levels of depression, anxiety, and stress symptoms. In the present study, Cronbach’s alpha coefficients were found to be 0.87 for depression, 0.84 for anxiety, and 0.83 for stress.

### Data collection procedure

An online survey form including the data collection instruments used in the study was developed using Google Forms and distributed to participants. Data were collected through social media platforms such as WhatsApp, Instagram, and LinkedIn, as well as through e-mail. Data collection was conducted between 1 June and 4 June 2026. The study was conducted in accordance with the approved study protocol, and participation was entirely voluntary. Participants were informed about the purpose and scope of the study and confidentiality principles through an informed consent form presented on the first page of the survey. No personally identifying information was collected, and survey responses were recorded anonymously. Participants indicated consent by responding to the statement “I voluntarily agree to participate in this study and provide my consent” with either “Yes” or “No,” and only those selecting “Yes” were included in the study. A total of 598 responses were collected. Of these, 10 responses were excluded because participants were older than 30 years, and 17 were excluded because the same response option was selected for every item on at least one scale, including reverse-worded items. Final analyses were conducted using data obtained from 571 participants. A distress protocol was also included at the end of the survey form to provide participants with information on available psychological support resources in case completing the survey caused discomfort or emotional distress.

### Data analysis

Data were analyzed using R software, and all statistical tests were two-tailed. Exact *p*-values were reported to three decimal places, whereas values below 0.001 were reported as *p* < 0.001. Preliminary analyses included descriptive statistics, internal consistency coefficients, Pearson correlations, and distributional checks. Skewness and kurtosis values within ±2 were considered acceptable for univariate normality (George and Mallery, 2010). Discriminant validity was assessed using item-level heterotrait–monotrait ratios of correlations (HTMT), with 0.85 used as the criterion (Henseler et al., 2015). Harman’s single-factor procedure was conducted using an unrotated principal-components analysis of the 65 substantive measurement items, excluding the three CTQ minimization/denial items. The variance explained by the first component was examined as a diagnostic indicator of potential common method bias (Podsakoff et al., 2003).

The hypotheses were tested using a regression-based conditional process analysis specified according to the logic of Hayes’ (2022) PROCESS Model 14. Childhood trauma was included in the mediator equation for vulnerable narcissism. Separate outcome equations were estimated for depression, anxiety, and stress, including childhood trauma, vulnerable narcissism, psychological resilience, and the vulnerable narcissism × psychological resilience interaction. Age and gender were included as covariates, and vulnerable narcissism and psychological resilience were mean-centered before the interaction term was calculated. Conditional indirect associations were estimated at low (−1 SD), mean, and high (+1 SD) levels of psychological resilience. At the mean level of resilience, the indirect association was also expressed as a fully standardized effect, and the proportion of the estimated total association accounted for by vulnerable narcissism was calculated as ab/(c’ + ab). Percentile bootstrap 95% confidence intervals based on 5,000 resamples were obtained for these estimates, and intervals excluding zero were considered statistically significant. Given the cross-sectional design, the indirect effects were interpreted as statistical associations rather than causal effects.

Regression coefficients were reported with HC3 heteroscedasticity-consistent standard errors and 95% confidence intervals, together with adjusted R^2^ values (Hayes and Cai, 2007; MacKinnon and White, 1985). Multicollinearity, regression assumptions, and influential observations were assessed using variance inflation factors, residual diagnostics, the Breusch–Pagan test, Ramsey’s RESET test, the Durbin–Watson statistic, leverage values, externally studentized residuals, and Cook’s distance. Model stability was examined by excluding observations exceeding the conservative Cook’s distance criterion of 4/*N*. Detailed diagnostic results are presented in Supplementary Table 4. Sensitivity analyses additionally adjusted the mediator and outcome equations for previous psychological support, current psychiatric medication use, educational level, and study/employment status. The primary models were also re-estimated after excluding participants scoring at the highest level on the CTQ minimization/denial index. Detailed results are reported in Supplementary Tables 1, 5.

Given the gender imbalance in the sample, exploratory gender interaction analyses were also conducted. Gender was coded as 1 = men and 2 = women. A childhood trauma × gender interaction was included in the mediator equation, whereas childhood trauma × gender, vulnerable narcissism × gender, psychological resilience × gender, and vulnerable narcissism × psychological resilience × gender interactions were entered simultaneously into each outcome equation. Continuous predictors involved in the interaction terms were mean-centered, HC3 standard errors were retained, and joint Wald tests were used to evaluate the gender interaction terms. Because the groups were unequal in size and the male subgroup was smaller, these analyses were treated as exploratory. Detailed coefficients are reported in Supplementary Table 3.

## Results

### Preliminary analyses

#### Sociodemographic characteristics

The sample consisted of 571 young adults (*M*_age = 21.99, SD = 3.14; range = 18–30), predominantly women (76.7%), single (90.7%), and university students (59.5%). Nearly half of the participants were students only (48.4%), whereas smaller proportions were working only (22.6%), both studying and working (19.4%), or neither in education nor employment (9.6%). Approximately one quarter (25.6%) reported previous psychological support, and 4.0% reported psychiatric medication use.

#### Descriptive statistics, reliability, and correlations

Descriptive statistics, internal consistency coefficients, and bivariate correlations were examined to provide a preliminary overview of the study variables and their interrelationships.

As shown in Table 1, internal consistency coefficients ranged from acceptable to excellent (α = 0.76–0.92). Skewness and kurtosis values were within ±2 for all variables, indicating no substantial departures from univariate normality.

**TABLE 1:** Descriptive statistics and internal consistency coefficients for the study variables.

Variable	*n*	*M*	SD	Median	Min	Max	Skewness	Kurtosis	α
Age	571	21.99	3.14	21.00	18.00	30.00	0.94	0.10	–
CTQ-33 total	571	46.61	14.43	43.00	30.00	109.00	1.33	1.73	0.92
Vulnerable narcissism	571	20.10	5.66	21.00	8.00	38.00	−0.11	−0.05	0.76
Psychological resilience	571	19.27	4.72	19.00	6.00	30.00	−0.21	0.10	0.79
Depression	571	5.42	4.26	5.00	0.00	21.00	0.87	0.59	0.87
Anxiety	571	4.59	3.95	4.00	0.00	20.00	0.98	0.83	0.84
Stress	571	6.30	4.15	6.00	0.00	21.00	0.69	0.29	0.83

α, Cronbach’s alpha. Skewness and kurtosis values were examined to assess univariate normality. Following George and Mallery (2010), values within ±2 were considered indicative of acceptable normality.

As shown in Table 2, childhood trauma was positively correlated with vulnerable narcissism (*r* = 0.289), depression (*r* = 0.516), anxiety (*r* = 0.456), and stress (*r* = 0.443). Psychological resilience was negatively correlated with vulnerable narcissism (*r* = −0.324), depression (*r* = −0.454), anxiety (*r* = −0.465), and stress (*r* = −0.425). Vulnerable narcissism was positively correlated with depression (*r* = 0.467), anxiety (*r* = 0.457), and stress (*r* = 0.481). The three psychological symptom domains were also strongly intercorrelated (*rs* = 0.734–0.771). All correlations were statistically significant at *p* < 0.001.

**TABLE 2:** Pearson correlations among the main study variables.

Variable	1	2	3	4	5	6
1. CTQ-33 total	1.000	–	–	–	–	–
2. Vulnerable narcissism	0.289	1.000	–	–	–	–
3. Psychological resilience	−0.195	−0.324	1.000	–	–	–
4. Depression	0.516	0.467	−0.454	1.000	–	–
5. Anxiety	0.456	0.457	−0.465	0.749	1.000	–
6. Stress	0.443	0.481	−0.425	0.771	0.734	1.000

All bivariate correlations were significant at *p* < 0.001. Values represent Pearson’s r coefficients.

#### Common method bias and discriminant validity

The unrotated principal-components analysis yielded 12 components with eigenvalues greater than 1. The first component accounted for 24.7% of the total variance, indicating that no single general component dominated the covariance among the measurement items. Because Harman’s single-factor procedure is a limited post hoc diagnostic, this finding was not interpreted as ruling out common method bias.

Heterotrait–monotrait ratios of correlations analyses supported the discriminant validity of vulnerable narcissism and psychological resilience in relation to the psychological symptom domains. The HTMT ratio between vulnerable narcissism and psychological resilience was 0.470. The ratios of vulnerable narcissism with depression, anxiety, and stress were 0.572, 0.571, and 0.604, respectively, whereas the corresponding ratios for psychological resilience were 0.549, 0.571, and 0.527. These values were below the conservative threshold of 0.85. In contrast, the HTMT ratios among depression, anxiety, and stress ranged from 0.875 to 0.906, indicating substantial overlap among the DASS-21 domains. These outcomes were therefore interpreted as closely related internalizing symptom domains rather than fully independent constructs. The complete HTMT matrix is presented in Supplementary Table 2.

### Testing the conditional process model

The regression coefficients, confidence intervals, and model-fit statistics for the mediator and outcome equations are presented in Table 3.

**TABLE 3:** Regression coefficients, confidence intervals, and model fit statistics for the conditional process models.

Model	Predictor	B	SE	95% CI	β	*P*	R^2^	Adjusted R^2^
VNS	CTQ-33 total	0.115	0.017	[0.081, 0.148]	0.292	<0.001	0.091	0.086
	Age	−0.072	0.074	[−0.218, 0.074]	−0.040	0.334	–	–
	Gender	0.897	0.529	[−0.142, 1.936]	0.067	0.090	–	–
Depression	CTQ-33 total	0.113	0.013	[0.088, 0.138]	0.382	<0.001	0.452	0.446
	Vulnerable narcissism	0.195	0.028	[0.140, 0.249]	0.259	<0.001	–	–
	Psychological resilience	−0.298	0.037	[−0.371, −0.225]	−0.285	<0.001	–	–
	VNS × BRS	−0.001	0.005	[−0.012, 0.009]	−0.008	0.794	–	–
	Age	−0.060	0.041	[−0.140, 0.020]	−0.044	0.144	–	–
	Gender	−0.116	0.298	[−0.702, 0.469]	−0.012	0.696	–	–
Anxiety	CTQ-33 total	0.088	0.012	[0.064, 0.112]	0.322	<0.001	0.414	0.407
	Vulnerable narcissism	0.178	0.027	[0.126, 0.231]	0.257	<0.001	–	–
	Psychological resilience	−0.289	0.034	[−0.357, −0.222]	−0.300	<0.001	–	–
	VNS × BRS	−0.007	0.006	[−0.018, 0.005]	−0.040	0.272	–	–
	Age	−0.018	0.039	[−0.095, 0.059]	−0.014	0.644	–	–
	Gender	0.351	0.283	[−0.204, 0.907]	0.038	0.215	–	–
Stress	CTQ-33 total	0.086	0.012	[0.062, 0.110]	0.301	<0.001	0.404	0.397
	Vulnerable narcissism	0.222	0.028	[0.167, 0.277]	0.304	<0.001	–	–
	Psychological resilience	−0.267	0.039	[−0.343, −0.190]	−0.263	<0.001	–	–
	VNS × BRS	−0.002	0.005	[−0.012, 0.007]	−0.012	0.673	–	–
	Age	−0.105	0.040	[−0.182, −0.027]	−0.079	0.008	–	–
	Gender	−0.369	0.332	[−1.020, 0.282]	−0.038	0.266	–	–

B, unstandardized regression coefficient; SE, HC3 heteroscedasticity-consistent standard error; CI, confidence interval; β, standardized regression coefficient; VNS, vulnerable narcissism; BRS, psychological resilience. Vulnerable narcissism and psychological resilience were mean-centered before calculating the interaction term. Gender was coded as 1, men and 2, women. Exact *p*-values are reported to three decimal places; values smaller than 0.001 are reported as *p* < 0.001.

As indicated in Table 3, childhood trauma was positively associated with depression, B = 0.113, 95% CI [0.088, 0.138], *p* < 0.001; anxiety, B = 0.088, 95% CI [0.064, 0.112], *p* < 0.001; and stress, B = 0.086, 95% CI [0.062, 0.110], *p* < 0.001. Thus, H1 was supported. Childhood trauma was also positively associated with vulnerable narcissism, B = 0.115, 95% CI [0.081, 0.148], *p* < 0.001. Vulnerable narcissism was positively associated with depression, B = 0.195, 95% CI [0.140, 0.249], *p* < 0.001; anxiety, B = 0.178, 95% CI [0.126, 0.231], *p* < 0.001; and stress, B = 0.222, 95% CI [0.167, 0.277], *p* < 0.001. These associations were subsequently examined within the indirect-association analysis. The mediator equation accounted for 9.1% of the variance in vulnerable narcissism (adjusted R^2^ = 0.086). The outcome models accounted for 45.2% of the variance in depression (adjusted R^2^ = 0.446), 41.4% of the variance in anxiety (adjusted R^2^ = 0.407), and 40.4% of the variance in stress (adjusted R^2^ = 0.397). Conditional indirect associations and indices of moderated mediation are presented in Table 4.

**TABLE 4:** Conditional indirect effects and indices of moderated mediation.

Outcome	Effect	B	LLCI	ULCI
	Indirect effect at low BRS (−1 SD)	0.023	0.013	0.035
Depression	Indirect effect at mean BRS	0.022	0.014	0.032
	Indirect effect at high BRS (+1 SD)	0.022	0.013	0.032
	Index of moderated mediation	0.000	−0.001	0.001
	Indirect effect at low BRS (−1 SD)	0.024	0.013	0.038
Anxiety	Indirect effect at mean BRS	0.021	0.013	0.030
	Indirect effect at high BRS (+1 SD)	0.017	0.010	0.027
	Index of moderated mediation	−0.001	−0.002	0.000
	Indirect effect at low BRS (−1 SD)	0.026	0.015	0.040
Stress	Indirect effect at mean BRS	0.026	0.016	0.037
	Indirect effect at high BRS (+1 SD)	0.025	0.015	0.036
	Index of moderated mediation	0.000	−0.001	0.001

B values represent unstandardized conditional indirect effects. Bootstrap, 5,000 resamples; LLCI, lower limit of the 95% bootstrap confidence interval; ULCI, upper limit of the 95% bootstrap confidence interval. Fully standardized indirect effects and proportions of the estimated total associations accounted for were calculated at the mean level of psychological resilience and are reported in the accompanying text.

As indicated in Table 4, childhood trauma showed statistically significant indirect associations with depression, anxiety, and stress through vulnerable narcissism at low, mean, and high levels of psychological resilience. At the mean level of resilience, the indirect association was 0.022 for depression, 0.021 for anxiety, and 0.026 for stress, with none of the bootstrap confidence intervals including zero. The fully standardized indirect association was 0.075 for depression (95% bootstrap CI [0.046, 0.107]), 0.075 for anxiety (95% bootstrap CI [0.045, 0.109]), and 0.088 for stress (95% bootstrap CI [0.055, 0.126]). Vulnerable narcissism accounted for an estimated 16.4% of the total association between childhood trauma and depression (95% bootstrap CI [10.0%, 24.0%]), 18.9% of the association with anxiety (95% bootstrap CI [11.2%, 28.9%]), and 22.8% of the association with stress (95% bootstrap CI [13.7%, 34.5%]). These findings supported H2, although the indirect associations were modest in magnitude. As further indicated in Table 3, psychological resilience was negatively associated with depression, B = −0.298, 95% CI [−0.371, −0.225], *p* < 0.001; anxiety, B = −0.289, 95% CI [−0.357, −0.222], *p* < 0.001; and stress, B = −0.267, 95% CI [−0.343, −0.190], *p* < 0.001. However, the vulnerable narcissism × psychological resilience interaction was non-significant for depression, B = −0.001, 95% CI [−0.012, 0.009], *p* = 0.794; anxiety, B = −0.007, 95% CI [−0.018, 0.005], *p* = 0.272; and stress, B = −0.002, 95% CI [−0.012, 0.007], *p* = 0.673. Therefore, H3 was not supported. Table 4 further shows that the indices of moderated mediation were non-significant for depression, index = 0.000, 95% bootstrap CI [−0.001, 0.001]; anxiety, index = −0.001, 95% bootstrap CI [−0.002, 0.000]; and stress, index = 0.000, 95% bootstrap CI [−0.001, 0.001]. Because each confidence interval included zero, H4 was not supported.

#### Regression diagnostics

No problematic multicollinearity was detected: variance inflation factors ranged from 1.00 to 1.25 across the models. Ramsey’s RESET tests did not indicate material functional-form misspecification (*p*s = 0.067–0.910), and Durbin–Watson statistics ranged from 1.92 to 2.01. The Breusch–Pagan test was non-significant for the mediator equation but significant for the depression, anxiety, and stress equations. Accordingly, HC3 heteroscedasticity-consistent standard errors were retained for all regression models. Maximum Cook’s distance values ranged from 0.064 to 0.102, and no observation approached the conventional value of 1. Re-estimating the models after excluding observations exceeding the conservative 4/N criterion did not alter the direction or statistical significance of the hypothesized main associations and interaction terms. Detailed diagnostic findings are presented in Supplementary Table 4.

#### Sensitivity analyses

The models were re-estimated after 73 participants scoring at the highest level on the CTQ-33 minimization index were excluded (*n* = 498). Childhood trauma and vulnerable narcissism remained positively associated with the symptom domains, psychological resilience remained negatively associated with the outcomes, and the vulnerable narcissism × psychological resilience interaction terms remained non-significant. The pattern and interpretation of the primary findings therefore remained unchanged. Detailed coefficients are presented in Supplementary Table 5.

#### Adjustment for additional sociodemographic and clinical covariates

A second sensitivity analysis additionally adjusted the mediator and outcome equations for previous psychological support, psychiatric medication use, educational level, and study/employment status. The association between childhood trauma and vulnerable narcissism remained significant, B = 0.104, SEHC3 = 0.017, *p* < 0.001. Childhood trauma, vulnerable narcissism, and psychological resilience also remained significantly associated with all three symptom domains. The vulnerable narcissism × psychological resilience interaction remained non-significant for depression (*p* = 0.924), anxiety (*p* = 0.547), and stress (*p* = 0.955). The covariate-adjusted models accounted for 46.7% of the variance in depression, 44.3% of the variance in anxiety, and 42.3% of the variance in stress. Thus, adjustment for these additional sociodemographic and clinical covariates did not alter the pattern or interpretation of the primary findings. Full estimates are presented in Supplementary Table 1.

#### Exploratory analyses by gender

Exploratory interaction analyses did not provide evidence that the principal associations differed by gender. Gender did not significantly moderate the association between childhood trauma and vulnerable narcissism, B = 0.017, SEHC3 = 0.038, *p* = 0.659. Joint Wald tests of the gender interaction terms were also non-significant for depression, χ^2^(4) = 2.19, *p* = 0.700; anxiety, χ^2^(4) = 2.42, *p* = 0.660; and stress, χ^2^(4) = 1.80, *p* = 0.773. None of the individual gender interaction terms in the outcome models reached statistical significance (*p*s ≥ 0.205). The observed pattern of associations was broadly similar for women and men in this sample. However, because the gender groups were unequal in size, these findings were treated as exploratory and were not interpreted as evidence of gender invariance. Detailed results are presented in Supplementary Table 3.

## Discussion

This study examined the mediating role of vulnerable narcissism and the moderating role of psychological resilience in the relationship between childhood trauma and symptoms of depression, anxiety, and stress among young adults. Childhood trauma was positively associated with all three symptom domains. Vulnerable narcissism partially mediated these associations, whereas psychological resilience did not moderate the association between vulnerable narcissism and psychological distress. H1 and H2 were therefore supported, while H3 and H4 were not supported. These findings reflect the relationships estimated within the specified cross-sectional model and should not be interpreted as evidence of a developmental or causal sequence.

### The association between childhood trauma and psychological symptoms

The first hypothesis was supported: childhood trauma was significantly and positively associated with symptoms of depression, anxiety, and stress. This finding is consistent with meta-analytic evidence linking multiple adverse childhood experiences with adult mental health problems (Hughes et al., 2017) and specific forms of childhood maltreatment with adult depression (Mandelli et al., 2015). It is also consistent with recent findings showing significant associations between adverse childhood experiences and depression, anxiety, and stress among Turkish emerging adults (Girgin et al., 2025). Similar findings have been reported among Turkish university students, where childhood emotional abuse and neglect were associated with depressive symptoms through statistical indirect pathways involving insecure attachment, guilt, and loneliness (Guler, 2022). Previous studies also point to several emotional and interpersonal processes that may help explain these associations. Emotion regulation difficulties statistically mediated the associations of childhood trauma with depression and post-traumatic stress symptoms among Turkish women (Alpay et al., 2017). Among Chinese college students, perceived social support and emotion regulation difficulties operated as separate and sequential statistical mediators of the association between childhood maltreatment and depression (Huang et al., 2026). In a Turkish adult sample, negative beliefs about emotion and psychological inflexibility similarly mediated the associations of childhood maltreatment with depression, anxiety, and stress (Taşören, 2023). Attachment theory and schema theory provide complementary frameworks for interpreting these findings by linking adverse developmental experiences with insecure relational expectations and maladaptive beliefs about the self, others, and emotions (Guler, 2022; Young et al., 2003). Consistent with this interpretation, maladaptive cognitive emotion-regulation strategies statistically mediated the associations of childhood trauma with current depression and anxiety severity in a clinical sample (Huh et al., 2017).

Evidence from a sample of Turkish nurses further showed that emotional abuse and emotional neglect were independently associated with depression, anxiety, and stress scores (Gürsoy and Mechmet, 2023). Longitudinal findings indicate that childhood trauma is associated with greater persistence and chronicity of depressive and comorbid depressive-anxiety disorders (Hovens et al., 2012), as well as with elevated depression and anxiety symptom levels that remain evident across extended follow-up periods (Kuzminskaite et al., 2022). The present results are therefore consistent with a broader body of evidence linking childhood trauma with psychological difficulties. Nevertheless, because the present study is cross-sectional, childhood trauma should be interpreted as a concurrent statistical correlate of psychological symptoms rather than as a prospectively established causal risk factor.

### The partial mediating role of vulnerable narcissism

The second hypothesis was supported within the specified cross-sectional mediation model. Vulnerable narcissism partially mediated the associations of childhood trauma with depression, anxiety, and stress. However, the fully standardized indirect effects were modest in magnitude, ranging from 0.075 to 0.088. The indirect associations through vulnerable narcissism corresponded to approximately 16%–23% of the estimated total associations, while the direct associations between childhood trauma and all three symptom domains remained significant. Vulnerable narcissism should therefore be interpreted as a modest partial mediator rather than as a dominant or comprehensive explanation of the associations between childhood trauma and the three symptom domains.

This pattern is broadly consistent with research showing associations between childhood maltreatment and narcissistic vulnerability. Although the outcome was binge-watching rather than internalizing symptoms, Verrastro et al. (2024) similarly reported significant indirect associations between childhood emotional abuse and binge-watching through vulnerable narcissism and emotion dysregulation in a sample of 1,082 emerging adults. Among adult women, childhood maltreatment was more strongly associated with vulnerable narcissism than with grandiose narcissism, with dissociation statistically mediating both associations (Bertele et al., 2022). Blundell and van Doorn (2025) similarly found that vulnerable narcissism partially mediated the association between childhood abuse and neglect and prodromal psychosis in a cross-sectional adult sample. In mental health outpatients, perceived childhood emotional neglect was associated with depressive and generalized anxiety symptoms through statistically significant indirect associations involving narcissistic vulnerability, both independently and sequentially with shame (Kealy et al., 2023). Concurrent associations among childhood trauma, vulnerable narcissism, and social anxiety have also been reported, although vulnerable narcissism was not examined as a mediator in that study (Gökçay et al., 2024).

Related findings indicate that these associations may vary according to population and developmental context. Ensink et al. (2017) found that vulnerable narcissism statistically mediated the associations of maltreatment with internalizing and externalizing difficulties among girls aged 14–21 years, whereas comparable maltreatment-narcissism associations were not observed among boys. In a Turkish young adult sample, Şar and Türk-Kurtça (2021) found no direct association between childhood trauma and vulnerable narcissism. Instead, vulnerable narcissism was associated with preoccupied attachment and depression, while depression was associated with emotional neglect and overprotection-overcontrol. Overprotection-overcontrol has also been validated as an additional childhood adversity dimension within the expanded Turkish Childhood Trauma Questionnaire (Şar et al., 2021). These findings suggest that the association between childhood adversity and narcissistic vulnerability may involve both direct and indirect statistical patterns rather than a single uniform developmental pathway.

Vulnerable narcissism is characterized by interpersonal hypersensitivity, insecurity, feelings of inadequacy, contingent self-esteem, and heightened dependence on recognition or approval from others (Hendin and Cheek, 1997; Miller et al., 2011; Pincus et al., 2009). Otway and Vignoles (2006) found that recollections of parental coldness and excessive parental admiration were associated with both overt and covert forms of narcissism, while covert narcissism was additionally associated with attachment anxiety. These findings provide a basis for understanding vulnerable narcissism as a personality-based pattern involving fragile self-regulation and insecure relational expectations. Its relevance to psychological symptoms is further supported by evidence from psychiatric outpatients showing that vulnerable narcissism was indirectly associated with depressive symptoms through unprocessed emotion (Kealy et al., 2020). The available evidence therefore positions narcissistic vulnerability as a potentially relevant self-related personality process within the broader relationship between childhood adversity and psychological symptoms.

These theoretical interpretations should nevertheless be distinguished from what can be concluded from the present findings. Vulnerable narcissism and psychological symptoms were measured concurrently, while childhood trauma was assessed retrospectively. The significant indirect effects are consistent with partial statistical mediation, but they do not establish that vulnerable narcissism developed as a consequence of childhood trauma or subsequently produced depression, anxiety, and stress. Alternative temporal orderings and reciprocal relationships remain possible. Longitudinal research is needed to determine whether changes in vulnerable narcissism temporally account for the association between childhood trauma and subsequent symptoms of depression, anxiety, and stress.

### Psychological resilience as a direct correlate rather than a moderator

The third and fourth hypotheses were not supported. Psychological resilience was negatively associated with depression, anxiety, and stress, but neither its interaction with vulnerable narcissism nor the indices of moderated mediation were statistically significant. Although the conditional indirect associations through vulnerable narcissism were significant at low, mean, and high levels of resilience, their magnitudes did not differ significantly across these levels. Thus, resilience functioned statistically as a direct negative correlate of psychological symptoms rather than as a moderator of the association between vulnerable narcissism and these symptoms.

The proposed moderation was based on the distinction between compensatory and protective models of resilience. In a compensatory model, a promotive factor is independently associated with an outcome, whereas a protective factor modifies the strength of the association between a risk factor and an adverse outcome (Fergus and Zimmerman, 2005). The placement of resilience at the association between vulnerable narcissism and psychological symptoms was informed by evidence that narcissistic vulnerability is associated with shame, interpersonal distress, socially avoidant interpersonal problems, denial, and behavioral disengagement in response to stress (Fernie et al., 2016; Pincus et al., 2009). Vulnerable narcissism has also been negatively associated with several dimensions of resilience, particularly the capacity to recover following stressful disruption (Sękowski et al., 2023), and with mental toughness, while showing a positive association with perceived stress (Papageorgiou et al., 2019). Meta-analytic evidence further links vulnerable narcissism with negative mental health outcomes (Hou et al., 2025). However, these studies did not directly demonstrate that resilience, as assessed by the Brief Resilience Scale, attenuates the association between vulnerable narcissism and psychological symptoms. The present findings therefore do not support this specific theoretical extension.

The observed pattern is more consistent with a compensatory than a protective model. Resilience has been conceptualized as a stable trajectory of healthy functioning following adversity (Bonanno, 2004), the perceived ability to recover from stress (Smith et al., 2008), the capacity of a dynamic system to adapt successfully to disturbances (Masten, 2014), and the influence of psychological characteristics within the broader stress process (Fletcher and Sarkar, 2013). These conceptualizations do not imply that resilience must moderate every association between risk and psychological symptoms. Meta-analytic evidence showing a negative association between resilience and neuroticism is also consistent with its general association with psychological adjustment (Oshio et al., 2018). Nevertheless, because the present study was cross-sectional, resilience should be described as a direct negative correlate rather than as a factor demonstrated to protect individuals from subsequent distress.

The absence of moderation may reflect the context-dependent nature of resilience. Cao et al. (2024) found that resilience moderated the association between a high-neglect profile and subsequent internalizing problems but not the associations involving other maltreatment profiles or externalizing problems. Wingo et al. (2010) similarly reported moderation for non-childhood trauma exposure but not for childhood abuse in relation to depressive symptoms. Buffering effects have been identified for childhood abuse and neglect in relation to depression among Chinese college students (Chang et al., 2021), for neuroticism in relation to depression among Chinese adolescents exposed to emotional maltreatment (Hu et al., 2025), and for particular associations between adverse childhood experiences and mental health outcomes among Kenyan youth (Mutiso et al., 2025). These differences suggest that moderation by resilience may depend on the population, adversity, outcome, resilience measure, and statistical pathway examined. The general recovery capacity assessed by the Brief Resilience Scale may therefore be associated with lower symptom levels without modifying the more specific association between vulnerable narcissism and symptoms of depression, anxiety, and stress. Alternative placements of resilience also remain plausible. Zheng et al. (2022) identified a statistical chain indirect association involving childhood trauma, resilience, daily stress, and depression. Liu et al. (2026) found that perceived stress statistically mediated the association between adverse childhood experiences and comorbid anxiety and depression, while resilience moderated the association between adverse childhood experiences and perceived stress. These alternative models were not tested in the present study and cannot be inferred from the non-significant moderation findings. Future longitudinal research should compare these competing specifications using temporally separated measurements. On the basis of the present cross-sectional results, resilience and vulnerable narcissism should be regarded as related but distinct correlates of symptoms of depression, anxiety, and stress.

### Limitations

Because the study employed a cross-sectional design, causal and temporal inferences cannot be drawn, and the proposed ordering of childhood trauma, vulnerable narcissism, and psychological symptoms cannot be established from the present data. Reverse or reciprocal associations also remain plausible; for example, current symptom levels may have influenced self-evaluations and retrospective reports of childhood experiences. A related concern arises from assessing all variables through self-report instruments administered in a single online survey, with childhood trauma measured retrospectively. Common method variance, shared negative affectivity, recall bias, social desirability, and response-style effects may therefore have affected the observed associations. Although Harman’s single-factor diagnostic did not indicate that one factor accounted for most of the covariance, this procedure cannot exclude common method bias. Clarifying the direction and robustness of the associations will require longitudinal designs incorporating temporally separated measurements and independent data sources, such as clinician or informant reports.

Measurement-related limitations should also be considered. Confirmatory factor analyses were not conducted to re-examine the structures of the instruments in the present sample. Published Turkish validation studies support their established factor structures, and the sample-specific HTMT results supported the discriminant validity of vulnerable narcissism and psychological resilience in relation to the symptom domains. Nevertheless, HTMT ratios among depression, anxiety, and stress ranged from 0.875 to 0.906, indicating substantial overlap. These outcomes should therefore be regarded as closely related symptom domains rather than fully independent constructs. Future research should test the measurement models using estimators appropriate for ordinal data and examine measurement invariance across gender and other demographic groups. Questions of generalizability also arise from convenience sampling, the predominance of women (76.7%), the concentration of participants in university education, and the non-clinical nature of the sample. In addition, the smaller number of men (*n* = 133) reduced the power and precision of the exploratory gender comparisons. Non-significant interactions cannot, in this context, be taken as evidence of equivalence between women and men. More balanced samples drawn from educational, occupational, community, and clinical settings would help determine whether the findings extend to other young adult populations.

A final limitation concerns the use of the CTQ-33 total score. Although this approach was consistent with the construct-level focus of the model, it precluded conclusions about the relative or unique associations of emotional, physical, and sexual abuse; emotional and physical neglect; and overprotection–overcontrol. Accordingly, references to specific forms of childhood adversity in the theoretical discussion should not be interpreted as subtype-specific findings of the present study. Comparative analyses of the individual trauma domains are needed to clarify their distinct associations with vulnerable narcissism and psychological symptoms. The scope of the model was further limited by its focus on psychological resilience without the inclusion of other potentially relevant variables, such as social support, attachment patterns, emotion regulation, and self-esteem. Despite adjustment for several sociodemographic and clinical characteristics in the sensitivity analyses, residual confounding related to symptom history, treatment experiences, socioeconomic adversity, and other personality characteristics can not be excluded.

## Conclusion

Although statistically significant, the indirect associations between childhood trauma and symptoms of depression, anxiety, and stress through vulnerable narcissism were modest, while the corresponding direct associations remained significant. Vulnerable narcissism is therefore best understood as one self-related correlate of the trauma–distress relationship rather than as a comprehensive explanation. Moreover, the cross-sectional design does not permit the observed pattern to be interpreted as a temporal, causal, or developmental process. Despite being independently associated with lower levels of depression, anxiety, and stress, psychological resilience did not moderate the vulnerable narcissism–symptom associations, and the corresponding indices of moderated mediation were likewise non-significant. Rather than supporting a buffering role, this pattern was more consistent with a direct promotive association. Clinical interpretation must nevertheless remain cautious because the sample was non-clinical, and no specific recommendations for assessment or intervention can be derived from these findings. Prospective studies involving more diverse and clinical samples are needed to clarify the direction of the observed associations and examine alternative positions of resilience within the model.

## Data Availability

The raw data supporting the conclusions of this article will be made available by the corresponding author upon reasonable request, without undue reservation. Requests to access the datasets should be directed to fatihalpcebeci@gmail.com.

## References

[B1] Alpay E. H. Aydın A. Bellur Z. (2017). The mediating role of the emotion regulation difficulties on the relationship between childhood traumas, posttraumatic stress symptoms and depression. *Turk. J. Clin. Psychiatry* 20, 218–226. 10.5505/KPD.2017.98852

[B2] Arnett J. J. (2000). Emerging adulthood: A theory of development from the late teens through the twenties. *Am. Psychol.* 55, 469–480. 10.1037/0003-066X.55.5.469 10842426

[B3] Benzi I. M. A. Fontana A. Carone N. Locati F. Parolin L. Ensink K. (2026). Pathology of the self: A network analysis of personality functioning, narcissistic vulnerability, mentalizing, and epistemic trust across trauma profiles. *Borderline Pers. Disord. Emot. Dysregulation* 13:7. 10.1186/s40479-026-00335-5 41680886 PMC13005497

[B4] Bernstein D. P. Fink L. Handelsman L. Foote J. Lovejoy M. Wenzel K. et al. (1994). Initial reliability and validity of a new retrospective measure of child abuse and neglect. *Am. J. Psychiatry* 151, 1132–1136. 10.1176/ajp.151.8.1132 8037246

[B5] Bertele N. Talmon A. Gross J. J. (2022). Childhood maltreatment and narcissism: The mediating role of dissociation. *J. Interpersonal Violence* 37, N9525–N9547. 10.1177/0886260520984404 33356780

[B6] Blundell R. van Doorn G. (2025). Childhood abuse and prodromal psychosis: The mediating roles of grandiose and vulnerable narcissism. *Psychosis* 17, 252–263. 10.1080/17522439.2024.2434515

[B7] Bonanno G. A. (2004). Loss, trauma, and human resilience: Have we underestimated the human capacity to thrive after extremely aversive events? *Am. Psychol.* 59, 20–28. 10.1037/0003-066X.59.1.20 14736317

[B8] Büyüköztürk Ş Kılıç-Çakmak E. Akgün ÖE. Karadeniz Ş Demirel F. (2009). *Eğitimde bilimsel araştırma yöntemleri.* Ankara: Pegem Akademi. Turkish.

[B9] Cao J. Xu X. Man X. Fu X. Shen Z. Wang S. (2024). Protective role of resilience on the associations between childhood maltreatment and internalising and externalising problems. *Stress Health* 40:e3300. 10.1002/smi.3300 37573535

[B10] Chang J. J. Ji Y. Li Y. H. Yuan M. Y. Su P. Y. (2021). Childhood trauma and depression in college students: Mediating and moderating effects of psychological resilience. *Asian J. Psychiatry* 65:102824. 10.1016/j.ajp.2021.102824 34479025

[B11] Coşkun R. Altunışık R. V. Yıldırım E. (2017). *Sosyal bilimlerde araştırma yöntemleri SPSS uygulamalı.* Sakarya: Sakarya Yayıncılık. Turkish.

[B12] Doğan T. (2015). Kısa psikolojik sağlamlık ölçeği’nin Türkçe uyarlaması: Geçerlik ve güvenirlik çalışması. *J. Happiness Well-Being* 3, 93–102. Turkish

[B13] Ensink K. Chrétien S. Normandin L. Bégin M. Daigle D. Fonagy P. (2017). Pathological narcissism in adolescents: Relationships with childhood maltreatment and internalizing and externalizing difficulties. *Adolesc. Psychiatry* 7, 300–314. 10.2174/2210676608666180119165731

[B14] Fergus S. Zimmerman M. A. (2005). Adolescent resilience: A framework for understanding healthy development in the face of risk. *Annu. Rev. Public Health* 26, 399–419. 10.1146/annurev.publhealth.26.021304.144357 15760295

[B15] Fernie B. A. Fung A. Nikčević A. V. (2016). Different coping strategies amongst individuals with grandiose and vulnerable narcissistic traits. *J. Affect. Disord.* 205, 301–305. 10.1016/j.jad.2016.08.009 27552594

[B16] Fletcher D. Sarkar M. (2013). Psychological resilience: A review and critique of definitions, concepts, and theory. *Eur. Psychol.* 18, 12–23. 10.1027/1016-9040/a000124

[B17] Gao S. Yu D. Assink M. Chan K. L. Zhang L. Meng X. (2024). The association between child maltreatment and pathological narcissism: A three-level meta-analytic review. *Trauma Violence Abuse* 25, 275–290. 10.1177/15248380221147559 36651026

[B18] George D. Mallery P. (2010). *SPSS for Windows Step by Step: A Simple Guide and Reference 17.0 Update*, 10th Edn. Boston: Pearson.

[B19] Girgin Ç Kesici Ş Ak M. (2025). The relationships between adverse childhood experiences (ACEs) and distress among emerging adults: The mediating role of resilience. *Child Abuse Rev.* 34:e70072. 10.1002/car.70072

[B20] Gökçay H. Çabuk K. Yazıcı Karabulut İ Sağaltıcı E. Belli H. (2024). Interconnections between childhood trauma, narcissistic vulnerability, and social anxiety disorder: A cross-sectional study. *Neuropsychiatric Invest.* 62, 94–99. 10.5152/NeuropsychiatricInvest.2024.24029

[B21] Guler D. (2022). Childhood psychological maltreatment and depressive symptoms: Parallel-serial mediating effects of certain psychological factors. *Curr. Psychol.* 41, 4183–4193. 10.1007/S12144-021-02182-9

[B22] Gürsoy M. Y. Mechmet F. C. (2023). Correlations between childhood trauma and depression, anxiety, and stress levels in nurses. *Arch Psychiatr. Nurs.* 45, 164–168. 10.1016/j.apnu.2023.06.018 37544694

[B23] Hayes A. F. (2022). *Introduction to Mediation, Moderation, and Conditional Process Analysis: A Regression-Based Approach.* New York, NY: Guilford Publications.

[B24] Hayes A. F. Cai L. (2007). Using heteroskedasticity-consistent standard error estimators in OLS regression: An introduction and software implementation. *Behav. Res. Methods* 39, 709–722. 10.3758/BF03192961 18183883

[B25] Hendin H. M. Cheek J. M. (1997). Assessing hypersensitive narcissism: A reexamination of Murray’s Narcism Scale. *J. Res. Pers.* 31, 588–599. 10.1006/jrpe.1997.2204

[B26] Henseler J. Ringle C. M. Sarstedt M. (2015). A new criterion for assessing discriminant validity in variance-based structural equation modeling. *J. Acad. Mark. Sci.* 43, 115–135. 10.1007/s11747-014-0403-8

[B27] Hou R. Li S. Miller J. D. Lynam D. R. Xiang Y. (2025). Weapon or armor? Unpacking the paradox of narcissism and self-reported mental health through a three-level meta-analysis. *J. Pers* Advance Online Publication. 10.1111/jopy.70044 41431170 PMC13540336

[B28] Hovens J. G. Giltay E. J. Wiersma J. E. Spinhoven P. Penninx B. W. Zitman F. G. (2012). Impact of childhood life events and trauma on the course of depressive and anxiety disorders. *Acta Psychiatr. Scand.* 126, 198–207. 10.1111/j.1600-0447.2011.01828.x 22268708

[B29] Hu Y. Fei J. Yue J. Gao R. Song Q. Zhao X. et al. (2025). Emotional abuse and neglect, depression: A moderated mediation model of neuroticism and psychological resilience. *Psychiatry Invest.* 22, 389–396. 10.30773/pi.2024.0108 40262788 PMC12022791

[B30] Huang H. Luo L. Wu H. Jiao B. Lin J. Shi Y. et al. (2026). The relationship between childhood maltreatment and depression in college students: The chain mediating effects of perceived social support and difficulties in emotion regulation. *J. Interpersonal Violence* 41, 5455–5478. 10.1177/08862605251355978 40720178

[B31] Hughes K. Bellis M. A. Hardcastle K. A. Sethi D. Butchart A. Mikton C. et al. (2017). The effect of multiple adverse childhood experiences on health: A systematic review and meta-analysis. *Lancet Public Health* 2, e356–e366. 10.1016/S2468-2667(17)30118-4 29253477

[B32] Huh H. J. Kim K. H. Lee H. K. Chae J. H. (2017). The relationship between childhood trauma and the severity of adulthood depression and anxiety symptoms in a clinical sample: The mediating role of cognitive emotion regulation strategies. *J. Affect. Disord.* 213, 44–50. 10.1016/j.jad.2017.02.009 28189964

[B33] Kealy D. Laverdière O. Pincus A. L. (2020). Pathological narcissism and symptoms of major depressive disorder among psychiatric outpatients: The mediating role of impaired emotional processing. *J. Nervous Mental Dis.* 208, 161–164. 10.1097/NMD.0000000000001114 31977828

[B34] Kealy D. Laverdière O. Cox D. W. Hewitt P. L. (2023). Childhood emotional neglect and depressive and anxiety symptoms among mental health outpatients: The mediating roles of narcissistic vulnerability and shame. *J. Mental Health* 32, 24–32. 10.1080/09638237.2020.1836557 33084445

[B35] Kuzminskaite E. Vinkers C. H. Milaneschi Y. Giltay E. J. Penninx B. W. J. H. (2022). Childhood trauma and its impact on depressive and anxiety symptomatology in adulthood: A 6-year longitudinal study. *J. Affect. Disord.* 312, 322–330. 10.1016/j.jad.2022.06.057 35760192

[B36] Liu X. Song Y. Li C. Liu X. Han M. Wang G. et al. (2026). Adverse childhood experiences and comorbid anxiety and depression among older adults: A moderated mediation model of perceived stress and resilience. *J. Affect. Disord.* 398:120935. 10.1016/j.jad.2025.120935 41422965

[B37] Lovibond P. F. Lovibond S. H. (1995). The structure of negative emotional states: comparison of the Depression anxiety stress scales (DASS) with the beck depression and anxiety inventories. *Behav. Res. Therapy* 33, 335–343. 10.1016/0005-7967(94)00075-u 7726811

[B38] MacKinnon J. G. White H. (1985). Some heteroskedasticity-consistent covariance matrix estimators with improved finite sample properties. *J. Econometr.* 29, 305–325. 10.1016/0304-4076(85)90158-7

[B39] Mandelli L. Petrelli C. Serretti A. (2015). The role of specific early trauma in adult depression: A meta-analysis of published literature. *Eur. Psychiatry* 30, 665–680. 10.1016/j.eurpsy.2015.04.007 26078093

[B40] Masten A. S. (2014). Global perspectives on resilience in children and youth. *Child Dev.* 85, 6–20. 10.1111/cdev.12205 24341286

[B41] Miller J. D. Hoffman B. J. Gaughan E. T. Gentile B. Maples J. Keith Campbell W. (2011). Grandiose and vulnerable narcissism: A nomological network analysis. *J. Pers.* 79, 1013–1042. 10.1111/j.1467-6494.2010.00711.x 21204843

[B42] Mutiso V. Ndetei D. Jeremiah E. Nyamai P. Walusaka S. Onyango V. et al. (2025). The moderating and mediating role of resilience in the relationship between adverse childhood experiences and depression, PTSD, and suicidality in Kenyan youth. *Glob. Mental Health* 12:e88. 10.1017/gmh.2025.10041 40809792 PMC12345062

[B43] Oshio A. Taku K. Hirano M. Saeed G. (2018). Resilience and big five personality traits: A meta-analysis. *Pers. Individ. Dif.* 127, 54–60. 10.1016/j.paid.2018.01.048

[B44] Otway L. J. Vignoles V. L. (2006). Narcissism and childhood recollections: A quantitative test of psychoanalytic predictions. *Pers. Soc. Psychol. Bull.* 32, 104–116. 10.1177/0146167205279907 16317192

[B45] Papageorgiou K. A. Gianniou F.-M. Wilson P. Moneta G. B. Bilello D. Clough P. J. (2019). The bright side of dark: Exploring the positive effect of narcissism on perceived stress through mental toughness. *Pers. Individ. Dif.* 139, 116–124. 10.1016/j.paid.2018.11.004

[B46] Pincus A. L. Ansell E. B. Pimentel C. A. Cain N. M. Wright A. G. C. Levy K. N. (2009). Initial construction and validation of the Pathological narcissism inventory. *Psychol. Assess.* 21, 365–379. 10.1037/a0016530 19719348

[B47] Podsakoff P. M. MacKenzie S. B. Lee J.-Y. Podsakoff N. P. (2003). Common method biases in behavioral research: A critical review of the literature and recommended remedies. *J. Appl. Psychol.* 88, 879–903. 10.1037/0021-9010.88.5.879 14516251

[B48] Şar V. Türk-Kurtça T. (2021). The vicious cycle of traumatic narcissism and dissociative depression among young adults: A trans-diagnostic approach. *J. Trauma Dissoc.* 22, 502–521. 10.1080/15299732.2020.1869644 33427111

[B49] Şar V. Necef I. Mutluer T. Fatih P. Türk-Kurtça T. (2021). A revised and expanded version of the turkish childhood trauma questionnaire (ctq-33): Overprotection-overcontrol as additional factor. *J. Trauma Dissoc.* 22, 35–51. 10.1080/15299732.2020.1760171 32437267

[B50] Sarıçam H. (2018). The psychometric properties of Turkish version of Depression anxiety stress scale-21 (DASS-21) in health control and clinical samples. *J. Cogn. Behav. Psychotherapy Res.* 7, 19–30. 10.5455/JCBPR.274847

[B51] Sekaran U. (2003). *Research Methods for Business: A Skill-Building Approach*, 4th Edn. Hoboken, NJ: John Wiley & Sons.

[B52] Sękowski M. Subramanian Ł Żemojtel-Piotrowska M. (2023). Are narcissists resilient? Examining grandiose and vulnerable narcissism in the context of a three-dimensional model of resilience. *Curr. Psychol.* 42, 2811–2819. 10.1007/s12144-021-01577-y

[B53] Şengül B. Z. Ünal E. Akça S. Canbolat F. Denizci M. Bastuğ G. (2015). Validity and reliability study for the Turkish adaptation of the Hypersensitive narcissism scale (HSNS). *Düşünen Adam J. Psychiatry Neurol. Sci.* 28, 231–241. 10.5350/DAJPN2015280306

[B54] Set Z. (2021). Mediating role of narcissism, vulnerable narcissism, and self-compassion in the relationship between attachment dimensions and psychopathology. *Alpha Psychiatry* 22, 147–152. 10.5455/apd.99551 36425445 PMC9590667

[B55] Smith B. W. Dalen J. Wiggins K. Tooley E. Christopher P. Bernard J. (2008). The brief resilience scale: Assessing the ability to bounce back. *Int. J. Behav. Med.* 15, 194–200. 10.1080/10705500802222972 18696313

[B56] Taşören A. B. (2023). Childhood maltreatment and emotional distress: The role of beliefs about emotion and psychological inflexibility. *Curr. Psychol.* 42, 13276–13287. 10.1007/s12144-021-02594-7 35039733 PMC8754519

[B57] Turkish Statistical Institute [TurkStat]. (2026). *Adrese dayalı nüfus kayıt sistemi sonuçları, 2025 [Address based population registration system results, 2025].* Turkish. Available online at: https://veriportali.tuik.gov.tr/tr/press/53899 (accessed February 9, 2026).

[B58] Verrastro V. Calaresi D. Giordano F. Saladino V. (2024). Vulnerable narcissism and emotion dysregulation as mediators in the link between childhood emotional abuse and binge watching. *Eur. J. Invest. Health Psychol. Educ.* 14, 2628–2641. 10.3390/ejihpe14100173 39452168 PMC11507281

[B59] Wang X. Yu L. Li J. (2023). Relationships between childhood maltreatment, attachment security, and features of narcissism: A network analysis. *Pers. Individ. Dif.* 200:111890. 10.1016/j.paid.2022.111890

[B60] Wingo A. P. Wrenn G. Pelletier T. Gutman A. R. Bradley B. Ressler K. J. (2010). Moderating effects of resilience on depression in individuals with a history of childhood abuse or trauma exposure. *J. Affect. Disord.* 126, 411–414. 10.1016/j.jad.2010.04.009 20488545 PMC3606050

[B61] Yıldız S. Yukay Yüksel M. (2024). The mediator role of depression, stress and anxiety in the relationship between childhood trauma experiences and psychological vulnerability. *Int. J. Psychol. Educ. Stud.* 11, 247–257. 10.52380/ijpes.2024.11.3.1330

[B62] Young J. E. Klosko J. S. Weishaar M. E. (2003). *Schema Therapy: A Practitioner’s Guide.* New York, NY: Guilford Press.

[B63] Zheng K. Chu J. Zhang X. Ding Z. Song Q. Liu Z. et al. (2022). Psychological resilience and daily stress mediate the effect of childhood trauma on depression. *Child Abuse Neglect.* 125:105485. 10.1016/j.chiabu.2022.105485 35026440

