# Supplementary material for: Childhood trauma and psychological symptoms in young adults: examining the mediating role of vulnerable narcissism and the moderating role of psychological resilience
Source: Front Behav Neurosci. 2026 Sep 16;20:1904564. doi: 10.3389/fnbeh.2026.1904564 (PMC13624158; doi:10.3389/fnbeh.2026.1904564)
Supplement: Supplementary file 1 [file Data_Sheet_1.docx]

**Supplementary Material**

**Supplementary Table S1. Sensitivity Analyses Adjusting for Additional Sociodemographic and Clinical Covariates**

| **Equation/outcome** | **CTQ-33, B (SEHC3)** | **VNS, B (SEHC3)** | **BRS, B (SEHC3)** | **VNS × BRS, B (SEHC3), p** | **R²** |
| --- | --- | --- | --- | --- | --- |
| Vulnerable narcissism | 0.104 (0.017)*** | — | — | — | .128 |
| Depression | 0.102 (0.012)*** | 0.191 (0.028)*** | −0.295 (0.037)*** | −0.0005 (0.005), .924 | .467 |
| Anxiety | 0.076 (0.011)*** | 0.177 (0.026)*** | −0.274 (0.035)*** | −0.0036 (0.006), .547 | .443 |
| Stress | 0.074 (0.012)*** | 0.218 (0.028)*** | −0.258 (0.039)*** | −0.0003 (0.005), .955 | .423 |

***Note.*** All models additionally controlled for age, gender, previous psychological support, psychiatric medication use, educational level, and study/employment status. Educational level and study/employment status were entered using dummy variables. Standard errors are HC3 heteroscedasticity-consistent estimates. CTQ = Childhood Trauma Questionnaire; VNS = Vulnerable Narcissism Scale; BRS = Brief Resilience Scale. ***p < .001.

**Supplementary Table S2. Heterotrait–Monotrait Ratios of Correlations Among Vulnerable Narcissism, Psychological Resilience, and the DASS-21 Symptom Domains**

| **Construct** | **1** | **2** | **3** | **4** | **5** |
| --- | --- | --- | --- | --- | --- |
| 1. Vulnerable narcissism | — |  |  |  |  |
| 2. Psychological resilience | .470 | — |  |  |  |
| 3. Depression | .572 | .549 | — |  |  |
| 4. Anxiety | .571 | .571 | .875 | — |  |
| 5. Stress | .604 | .527 | .906 | .879 | — |

**Note.** Values are heterotrait–monotrait ratios of correlations (HTMT). Values below .85 were interpreted as supporting discriminant validity. The values among the DASS-21 domains indicate substantial overlap among these closely related symptom dimensions.

**Supplementary Table S3. Exploratory Tests of Gender Differences in the Conditional Process Model**

| **Equation/outcome** | **Gender interaction** | **B** | **SE (HC3)** | **p** |
| --- | --- | --- | --- | --- |
| Vulnerable narcissism | Childhood trauma × Gender | 0.017 | 0.038 | .659 |
| Depression | Childhood trauma × Gender | −0.023 | 0.035 | .504 |
|  | Vulnerable narcissism × Gender | −0.041 | 0.061 | .500 |
|  | Psychological resilience × Gender | 0.001 | 0.100 | .994 |
|  | Vulnerable narcissism × Resilience × Gender | 0.005 | 0.013 | .714 |
| Anxiety | Childhood trauma × Gender | −0.017 | 0.026 | .525 |
|  | Vulnerable narcissism × Gender | −0.014 | 0.074 | .851 |
|  | Psychological resilience × Gender | 0.026 | 0.077 | .741 |
|  | Vulnerable narcissism × Resilience × Gender | 0.020 | 0.016 | .205 |
| Stress | Childhood trauma × Gender | −0.027 | 0.031 | .389 |
|  | Vulnerable narcissism × Gender | 0.040 | 0.081 | .619 |
|  | Psychological resilience × Gender | 0.060 | 0.095 | .530 |
|  | Vulnerable narcissism × Resilience × Gender | −0.007 | 0.016 | .658 |

**Note.** Gender was coded 1 = men and 2 = women. Continuous predictors were mean-centered. Standard errors are HC3 heteroscedasticity-consistent estimates. Joint Wald tests of the four gender interaction terms were nonsignificant for depression, χ²(4) = 2.19, p = .700; anxiety, χ²(4) = 2.42, p = .660; and stress, χ²(4) = 1.80, p = .773. Because the analyses were exploratory and the gender groups were unequal in size, nonsignificant interactions should not be interpreted as evidence of measurement or structural invariance.

**Supplementary Table S4. Regression Diagnostics and Influence Statistics**

| **Model** | **VIF range** | **RESET *p*** | **Breusch–Pagan** | **DW** | **Maximum Cook’s *D*** | **Cook’s *D* > 4/*N*** | **Absolute externally studentized residual > 3** |
| --- | --- | --- | --- | --- | --- | --- | --- |
| **Vulnerable narcissism** | 1.003–1.004 | .067 | χ²(3) = 1.62, *p* = .656 | 1.92 | 0.064 | 26 | 0 |
| **Depression** | 1.018–1.247 | .500 | χ²(6) = 29.21, *p* < .001 | 1.98 | 0.093 | 26 | 7 |
| **Anxiety** | 1.018–1.247 | .638 | χ²(6) = 43.47, *p* < .001 | 1.95 | 0.102 | 32 | 5 |
| **Stress** | 1.018–1.247 | .910 | χ²(6) = 14.11, *p* = .028 | 2.01 | 0.089 | 33 | 4 |

Note. VIF = variance inflation factor; RESET = Ramsey Regression Equation Specification Error Test; DW = Durbin–Watson statistic. Cook’s distance values exceeding 4/N were treated as a conservative screening criterion rather than an automatic exclusion rule. No observation had a Cook’s distance approaching 1. Sensitivity models excluding observations exceeding 4/N preserved the direction and statistical significance of all hypothesized main and interaction effects.

**Supplementary Table S5. Sensitivity Analyses After Excluding Participants with a CTQ Minimization Score of 3**

| **Outcome** | **CTQ-33 (B, p)** | **VNS (B, p)** | **BRS (B, p)** | **VNS × BRS (B, p)** |
| --- | --- | --- | --- | --- |
| Depression | 0.113, < .001 | 0.180, < .001 | -0.270, < .001 | 0.000, .942 |
| Anxiety | 0.089, < .001 | 0.183, < .001 | -0.260, < .001 | -0.005, .370 |
| Stress | 0.082, < .001 | 0.216, < .001 | -0.239, < .001 | -0.001, .905 |

*Note.* Coefficients shown were obtained after excluding 73 participants with a CTQ minimization score of 3 (n = 498).
